# Essays on Surgery and Midwifery; with Practical Observations

**Published:** 1822-09-01

**Authors:** 


*Essays on Surgery and Midwifery; with Practical Observations, and Select Cases.* By JAMES BARLOW, Surgeon. With Plates. Octavo. London, 1822.

(Second Analytical Article, concluded from page 77.)

IN a former Number we gave an analysis of the first part of the volume before us. We now proceed to the second, or obstetrical portion of the work. The first Essay embraces an enquiry into the various opinions respecting the management of the placenta. This subject is prefaced by a disquisition on "the *proximate* cause of the pain and difficulty attendant on parturition," which our author is more inclined to impute "to the habits of dissipation and voluptuousness, and the deterioration of physical strength, than to any *immediate* infliction at the fall." We think the term *proximate* is ill applied here, at least as far as relates to civilization and dissipation; for they can have acted only as *predisposing* causes to the pain and difficulty attendant on man's entry into the world. With her Creator's fiat that, "in sorrow should she bring forth," we have nothing to do here; but, of the fact that civilization and refinement *increase* the original difficulty, we can entertain no doubt; for the contrast between the upper and lower classes of society, and still more between the savage and civilized, is too manifest not to carry conviction to the mind of the most inattentive observer.

Hippocrates, in several parts of his writings, but especially in books "de Morbis Mulierum," and "de Superfœtatione," has touched upon the subject of the placenta. His observations are, as usual, full of absurdities. First he directs a number of remedies by the mouth, for expelling the secundines, as garlic, onions, oysters, castor, beet-root, rice, &c.* then he describes an awkward and puerile mode of pulling the cord.† Of a very different complexion are the directions of Celsus;—which, indeed, are followed to this hour. "*Medicus deinde sinistra manu leniter trahere umbilicum debet, ita, ne abrumpat, dextraque eum sequi usque ad eas, quas secundas vocant, quod velamentum infantis intus fuit; hisque ultimis apprehensis, venulas membranulasque omnes eadem ratione manu diducere a vulva, totumque illud extrahere, et si quid intus præterea concreti sanguinis remanet.*"—*Lib.* VII. *Cap.* XXIX. As the above directions for the extraction of the placenta have not been one whit improved upon during the last eighteen hundred years,‡ we shall not trouble our readers with any quotations from Ætius, Paulus Egineta, Avicenna, or others since his time. Great contrariety of opinion, indeed, has prevailed on this point, till within a very recent period—one party being advocates for immediate extraction, considering the placenta as an inanimate and extraneous substance, and supposing it must prove dangerous to the mother, if allowed to remain in the uterus after the birth—the other party confiding in the almost omnipotent resources of Nature, and leaving the work to her spontaneous efforts. Neither of these doctrines is to be implicitly followed.

"Impressed by a conviction of the efficacy of nature in most instances, I am led to believe, that when art is unnecessary, manual interference becomes injurious; this is manifested on no occasion more clearly, than when the membranes are artificially ruptured during parturition, which generally induces a premature suspension of uterine action, before its orifice is disposed to yield naturally. Protracted labour is thereby incurred, together with a train of subsequent evils, inseparably connected with the whole parturient and puerperal condition; hence it is evident, that no officious interposition should take place between the expulsive action of the uterus, and the advances of the head of the child in the passage, during the regular process of a natural labour, and where the cavity of the pelvis presents no barrier to its course. On all occasions, it is best to watch this guide attentively, to discern her procedure, to support and aid her

---

* De Morbis Mulierum, LXXIII.

† "Cæterum si secunda non facile excidat, maxime quidem ad fœtum appendere sinenda est, et puerpera velut super sella ventri exoneranda destinata, lasanum Græci vocant. Si tandem in altum exstructa sella, quo fœtus dependens gravitate sua secundam simul extrahat."—*De Superfœtatione*, LXXVII. V.

‡ Mr. Barlow says that Celsus claims the merit of being the first who introduced the hand for the extraction of the secundines; but we cannot find such claim in his writings.—*Ed.*

efforts when inefficient, and when assistance is not requisite, to withhold every unseasonable interference, and forbear to subvert her endeavours by any obtrusive or precipitate attempts either to hasten labour, or extract the placenta by the hand." 195.

Hæmorrhage, to a certain extent, after the expulsion of the fœtus, calls necessarily for manual aid in removing the placenta, and thereby enabling the uterus to contract; but unless some cause of this kind require aid, we should not be too officious.

"If the accoucheur, immediately on the termination of labour, and before having waited to ascertain the inadequate functions of the uterus, instantly begin to pull at the funis, or force his hand into that organ, and drag the placenta out hastily, he will frequently hazard the life of the mother, by inverting the uterus, or inducing hæmorrhage by a disruption of the cord, or partial separation of the cake from the uterine surface." 196.

When the efforts of Nature have been uninterrupted during labour, the interval between the birth of the child and the expulsion of the placenta, rarely exceeds a quarter of an hour—and should this period be surpassed, it is still more prudent to wait the recurrent action of the uterus, than have recourse to manual interposition, unless some urgent reason be present. The expulsion of the placenta, however, may almost always be greatly expedited by gently drawing the cord, and pressing the region of the uterus over the pubis. The afterbirth will often lie in the vagina, and come no farther without considerable traction by the cord, or manual assistance. We have known young men wait for some hours, with the placenta in such situation, under the impression that they had retained placenta to deal with. Whenever the insertion of the cord into the placenta can be felt with the point of the finger introduced along the cord, we may be sure that the secundines are clear of the uterus, and may be drawn away without fear.

But although the expulsion of the placenta is, in the great majority of cases, the work of Nature; it is to be acknowledged, that her powers are sometimes inadequate, and then artificial assistance becomes necessary. The chief difficulties connected with the present enquiry, are arranged by our author under the following heads;—

1. Atony of the uterus attended with flooding.
2. Irregular and spasmodic action of the uterus.
3. Morbid adhesion of the placenta.

1. *Atony and Flooding.* This state may be induced by dilating and irritating the os uteri and vagina to expedite labour—by rupturing the membranes—by confined and heated apartments—by imprudent instrumental aid—by rashly dragging the body down, without waiting for the uterine contractions.

After waiting some time, and no hæmorrhage occurring, the uterine efforts being evidently suspended, the woman should be placed for awhile in a horizontal position to appease the system, after which, a mild cordial should be given, and cool air admitted into the apartment, while friction and gentle pressure on the hypogastric region should be employed. If these means fail, one or two fingers should be cautiously introduced up the vagina, whilst the funis is gently pulled by the other hand. If the orifice of the uterus be found in a favourable condition, and the lobulated surface of the after-birth be within reach of the finger, though situated over the promontory of the superior aperture, its expulsion may generally be accomplished by pressure on the abdomen with one hand, and gentle traction of the funis, in the direction of the pelvic apertures, by the other, "at the same time slightly stimulating the uterus by passing the fore finger a few times around its orifice, or by grasping it from without and exciting it through the relaxed parietes of the abdomen." Our author cautions the practitioner not to leave any of the filamentous membranes either in the vagina or suspended at the vulva, as they will frequently produce constitutional irritation and fever, besides becoming offensive in a few days from putrefaction. Here Mr. Barlow states two cases of inversion of the uterus; in one, attended by a midwife, he found the woman dead, the uterus wholly inverted without the labia, and the placenta in part attached to that organ. This woman died of profuse hæmorrhage in the space of an hour from the birth of the child. In the other case, the pains had been very violent during labour, and the practitioner informed Mr. Barlow, that on a very slight traction of the funis after delivery of the fœtus, the whole volume of the uterus became instantly inverted.

"On inspecting the state of the parts, I observed the uterus lying without the vulva, and the placenta firmly attached to its fundus, which organs reached midway to the woman's knees: fortunately, their adhesion to each other was so complete, that little hæmorrhage had ensued, this may be accounted for, by the inversion taking place so soon after birth, and before the uterus was allowed to act and separate from the placenta. Upon reflecting a few moments on the nature of the case, and the impossibility of a body like this, the magnitude of which exceeded that of the head of the child, repassing through the neck of the uterus, I, without delay, grasped the whole substance between my hands and by uniform pressure, continued for a few minutes, the blood oozed out, and it became considerably diminished. I then separated the placenta from its attachment to the uterus, and effected its reposition, by placing the points of my fingers against the fundus, and pushing it through its neck, and the inverted vagina, till the whole hand had completely passed the superior aperture of the pelvis, and entered into the proper cavity. Little or no hæmorrhage followed, and in order to prevent a future descent, a piece of sponge was introduced up the vagina, and retained in this situation by means of a T bandage.

"On removing the plug, and passing my finger up the vagina the following day, I found the cavity of the pelvis nearly occupied by the partially inverted uterus, as it were in a strangulated state of its neck: notwithstanding this disastrous occurrence by the introduction of the hand, the organ was soon reverted to its natural situation, and the vagina filled as before; the sponge was removed and replaced once every day, or as often as it became offensive, and the woman during several days, strictly kept in a recumbent posture, a cooling plan of regimen was enjoined, the bowels kept open by means of aperient clysters, occasionally administered, and she completely recovered in a few weeks without any material interruption, or return of the complaint. It is lamentable to remark, that I have been consulted in a considerable number of cases of *inverted* and *prolapsed uteri*, of the chronic kind, which, on enquiry, I have traced to the mismanagement of the placenta, and it is probable, that such maladies are more frequent than at first view would be supposed in the present improved state of the art, though I suspect numbers are, from motives of female delicacy, screened from professional notice." 209.

The mode of reposition, when the placenta is adherent to the inverted uterus, as recommended by great authorities, is to return both organs at the same time without attempting separation. This precept, Mr. Barlow thinks, should be received with great caution, "as the bulk of the two protruded organs, when surcharged with blood, will present such resistance to the exertions of the accoucheur, that it cannot be effected without using great violence, and exposing the woman to imminent and unnecessary danger." The subsequent separation within the uterine cavity may again occasion inversion. The best plan, our author thinks, is "to compress the whole substance between the hands like a sponge, by which the blood will escape, and the uterus be diminished in size." By this procedure the placenta will be detached, and the reduction of the uterus itself facilitated.

On all occasions, when the hand is to be introduced for the extraction of the placenta, the contents of the bladder and rectum should be previously evacuated:—and our author thinks that it is more eligible, when either the placenta is to be removed or the fœtus turned, to introduce the hand with the palm towards the pubis.

"On whatever side the woman be laid, by this means the hand is with greater facility conducted to the feet of the fœtus or placenta, particularly in women with pendulous bellies where the uterus is pushed out of the axis of the apertures of the pelvis. The hand of the accoucheur thus conducted into the uterus will be better able to effect the extraction through the axis of the brim, cavity, or outlet of the pelvis, than when employed in a contrary direction." 211.

Here we should wait the efforts of the uterus, unless an alarming or insidious hæmorrhage be present. In the absence of these, and when the uterus assumes a state of permanent torpor, "we may with safety withhold these manual means, and even wait two or more hours before attempting extraction by artificial efforts."* During this interval, the accoucheur should occasionally ascertain the state of the os uteri, lest internal hæmorrhage or contraction of the cervix take place. Frictions and mild cordials should be employed. Syncope or hæmorrhage authorize the immediate introduction of the hand, which is to be moved about in the uterine cavity till the contractile nisus is restored, after which, the hand may be gradually withdrawn, together with any remaining portion of coagula or loose fragments of the membranes.

In uterine, as in other hæmorrhages, syncope is more alarming than dangerous, as it gives time for a coagulum to form in the mouths of the vessels. It is not easy, however, to determine the precise time when to give cordials, and to what extent, in such perplexing cases. Opium is not implicitly relied on by our author, in uterine hæmorrhage:—"nevertheless, it is a valuable adjunct in many affections of the uterus, particularly in convulsions, and irregular spasmodic contractions of its muscular fibres." In syncope attendant on flooding, he thinks it might prove a dangerous medicine.

Where there is reason to expect uterine hæmorrhage and placental retention, from previous occurrences of this kind in the same female, a broad bandage applied steadily, and tightened immediately on the termination of labour, will greatly support the existing laxity of the abdominal parietes, and probably prevent or lessen the hæmorrhage.

Placental adhesion may be known, Mr. B. observes, by gently pulling at the funis. "If there be an elastic resilient sensation communicated to the hand, it is a certain proof of such attachment." In a passive state of the uterus, with re-

---

* Dr. William Hunter's doctrines respecting the placenta, led to a very passive management of the after-birth, and we have no doubt that many lives were lost in consequence. We know that the placenta, whether in abortions or in full-timed births, if allowed to remain, will very generally prove fatal.—*Ed.*

tained placenta, there is sometimes an insidious hæmorrhage, in which case, our conduct must be governed by the quantity of blood lost in a given time.

"When such an occurrence takes place immediately after the delivery of the child, and continues without intermission, till its effects are obviously manifested on the general system by a weak fluttering pulse, pallid countenance, clammy sweats, cold extremities, fainting, with *tinnitus aurium*, &c. &c. the patient becomes exhausted and would soon expire if the symptoms were not instantly counteracted. In these alarming instances, the most prompt and decisive measures should be employed effectually to remove the contents of the womb, by promoting its muscular contractions in the way abovementioned." 219.

To aid our efforts in such perilous circumstances, our author has frequently witnessed the most powerful and salutary effects from an opiate glyster thrown up the rectum, composed of four or six ounces of cold water, and sixty or eighty minims of laudanum. An astringent injection, of the same temperature, may also be thrown up into the vagina or uterus, to moderate the discharge of blood. This latter injection may be composed of ten ounces of cold water, or of decoction of oak bark, and fifteen or twenty grains of alum, to be repeated at discretion. A recumbent posture, cool apartment, and quietude, should, of course, be insisted on. If these means fail, our author recommends half a grain of the superacetate of lead, and about the same quantity of opium, to be given every third or fourth hour:—or, ten grains of nitre, ten drops of tincture of digitalis, and fifteen of laudanum, with an ounce of infusion of roses, every three or four hours. In addition to these means, Mr. Barlow applies to the abdomen, cloths wrung out of solution of common salt in vinegar and water—or if ice can be procured, a bladder filled with this substance bruised, and laid on the same part. We have employed ice introduced into the vagina with benefit. It is not easy to form a prognosis in cases of uterine hæmorrhage.

"A woman may be cheerful and apparently bearing the loss of blood without danger, with a pretty strong pulse, not exceeding ninety strokes in the minute, and die instantly; while another may have a pallid ghastly countenance, attended with sickness, dimness of sight, coldness of the extremities, loss of memory, and pulse scarcely perceptible, and still recover.

"Another may have these alarming symptoms, and yet linger several weeks or months, and eventually die of dropsy." 223.

It is difficult to lay down determinate rules respecting the placenta. Mr. B. thinks it desirable, upon the whole, that the expulsion of the secundines should follow delivery in one or two hours, and that this space should be allowed, rather than removing the placenta by art, immediately after the birth of the child. A profuse hæmorrhage succeeding delivery, and before the exit of the placenta justifies the propriety, our author observes, of *removing the contents of the womb without delay.** For a number of minute observations on this point, we must refer to the work itself.

2. *Irregular or Spasmodic Action of the Uterus.* On this, as on the former head, our author is rather too diffuse, and has been very unskilful in the arrangement of his ideas, so as to compress them in as small a compass of language as possible. Indeed, one of Mr. Barlow's greatest faults, in regard to composition, is tautology and circumlocution.

The most frequent, irregular, and partial contractions of the cervix uteri, occur during or after abortions that happen about the middle period of pregnancy, and are sometimes accompanied with flooding. When they take place soon after the birth of the fœtus, they generally oppose the spontaneous expulsion of the placenta, in an inverse ratio to the advancement of the gestation, and nothing, Mr. B. observes, is more likely to excite the uncontrollable agency of this viscus, than the precipitate act of artificially rupturing the membranes, with the view of facilitating the progress of abortion. Mr. B. thinks, however, that unless hæmorrhage or convulsions attend placental retention in these premature periods of utero-gestation, "we need not be over solicitous about the deliverance of the placenta." Mr. B. prefers leaving it to be expelled by the efforts of Nature, which will generally be sufficient to effect this purpose in less than twenty-four hours after the birth of the fœtus. Should the patient, therefore, appear to be exhausted by a continued emission of blood, Mr. B. recommends, in the first place, palliative measures, as injections of cold water into the vagina, the ap-

* In this article, we have thought it better to give an analysis purely of the sentiments of an old and experienced practitioner, than mix them with the sentiments of other writers. We may be allowed here, however, to quote a short passage from the work of Burns, which is strong authority on the interesting subject under discussion.

"The retention of the placenta," says he, "is not the cause of the hæmorrhage, but a joint effect together with it, of the torpor of the uterus. Our primary object, therefore, is *not* to extract the placenta, but to excite the uterus to brisker action. How improper and dangerous then must it be to thrust the hand into the uterus, grasp the placenta, and bring it instantly away. By this practice, we are apt to injure the uterus, and certainly cannot rely upon it for checking the hæmorrhage." —*Third Ed. p.* 487.

plication of ice, and the medicines mentioned in the preceding section. Plugging the vagina and mouth of the uterus with lint, tow, or sponge, soaked in a strong solution of alum, and supported firmly by the T bandage, keeping the patient cool and quiet, is recommended by Mr. Barlow, in these cases. But flooding after delivery at the full period, renders this measure more doubtful; for the uterine parietes being more expanded and less disposed to contract than in cases of abortion, blood may accumulate in, and distend, the uterus, while the plug is in the vagina. This may be obviated, he thinks, by the application of counter-pressure uniformly continued on the hypogastric region, prior to the introduction of the plug, by means of a broad roller passed a few times round the body and over the lower part of the abdomen. In abortions at an early period, attended with hæmorrhage, where it would be impracticable to introduce the hand into the uterus, and where the discharge has been unabated for a long time, recourse should be had to opium in addition to the forementioned measures.

The hour-glass contraction of the uterus, our author observes, may be ascertained to extend its fundus upwards above the umbilicus, by the application of the hand on the hypogastric region—its structure will also appear to the touch as if it were encircled with a tight ligature leaving a transverse sulcated impression on the part. Our author has known the contraction to embrace the substance of the placenta, which is one of the most dangerous and perplexing species of uterine spasm.

"If the woman be not much affected with syncope, a strong opiate may be given to relax the spasm, and induce a corresponding action of the uterine fibres; after which the accoucheur may proceed to introduce his hand cautiously up the vagina uteri, taking care not to separate any portion of the placenta from its surface as it passes along, till it gets beyond the encircled part of that viscus, lest hæmorrhage be produced; and having gained access into the upper chamber, he may then commence the operation, and effect the separation from above downwards. By these means the fundus will have liberty to contract on the hand as it is withdrawn along with the placenta. If the extraction be attempted by commencing the operation near the cervix or lower cavity of the uterus, and before the spasm of the columna uteri be removed, an hæmorrhage will inevitably ensue, and the patient be thereby exposed to imminent danger in the interim, or during the removal of the remaining portion of the mass from the upper recess of the womb." 245.

In some instances, especially where the gestation has not been completed, the contraction is at the os uteri, where, like a purse, it embraces the funis, anterior to the placenta.

In this case, our author observes, there is fortunately little or no hæmorrhage, and it would be wrong to draw the funis or attempt the introduction of the hand. If flooding should succeed the relaxation of the spasm, then the accoucheur may have recourse to extraction at discretion. If the spasm continue, however, opium may be tried internally, and frictions over the uterine region, trusting eventually to time and the efforts of Nature, "before attempting to extract the placenta by manual efforts."

3. *Morbid Adhesion of the Placenta.* This is fortunately not a very frequent occurrence. The secundines, though not prone to disease, do sometimes take on a morbid structure, assuming a scirrhous or cartilaginous state, opposing a formidable obstacle to the separation after birth. Our author has, in more than one instance, traced these morbid adhesions to an injury inflicted on the uterine region during gestation. Under such afflicting events, the attempt at disunion should be made with great care and deliberation.

"To whatever extent such attachment exists, and after the accoucheur has waited the appropriate time for the efforts of nature without prospect of advantage, he may commence the operation by conveying one hand cautiously into the uterus, whilst the other is applied on the hypogastrium to stay that organ. If only a few lobes of the placenta be identified with the surface of the uterus, the extraction may generally be accomplished by beginning at the in-edges, and peeling off the morbid adhesions from the uterus, thus gradually separating the different portions in succession, till a complete disunion be attained, and the substance of the placenta being grasped in the hand, the whole may be brought forth without further trouble." 258.

Should the os uteri remain in a flaccid and distensible condition after such operation, it announces a more unfavourable result, and affords reason to dread fainting and flooding; to obviate which, the surgeon should pass his hand again into the uterus, and keep it in motion there till an evident contraction be perceptible to the touch, during which, cold air should be freely admitted into the apartment, a compress soaked in cold vinegar repeatedly applied to the abdomen, and the horizontal position, after removal of the hand, strictly enjoined.

In those unfortunate cases where a permanent and extensive union has taken place between the uterus and placenta, resisting every prudential attempt to break through the cohesive attachments, a difficult question arises, whether to hazard a separation of these organic adhesions by force, or abandon the expulsion to the powers of nature—"for while a rash interference is censurable, an implicit confidence in the powers of Nature may be equally improper." Our author observes that, in his experience, where fatal consequences attended protracted retention in one instance, rash and precipitate extraction proved fatal to many. Risk attends both measures. "If the whole, or even a portion of the placenta remain detached in the uterus for any undue space of time, putrefaction and absorption will generally take place, and its detention eventually prove a source of much evil—such as detention of the abdomen, fever, inflammation, fœtor of the lochia, constitutional irritation, diarrhœa, and not unfrequently death."

"Under these circumstances the accoucheur will yield to the lesser evil, by passing his hand into the uterus at frequent intervals, and scooping out any extraneous and offensive coagula from its abode, or otherwise by the more eligible method of antiseptic injections, as before mentioned." 261.

For some judicious observations on moles, polypi, and other unnatural growths in the uterus, we must refer to the volume itself.

At page 273 Mr. Barlow relates a case where the expulsion of the placenta preceded the delivery of the child. He was called to a woman in labour of her second child, the uterine pains being accompanied by a slight discharge of blood at intervals. The os tincæ was rigidly contracted. Our author left her, but next morning he was summoned, and found her seated on a chair in a state of great alarm, being informed by the attendants that a profuse discharge of blood succeeded every pain. On requesting her to go to bed, she was seized with a violent pain, which instantly expelled the placenta, and disparted the funis about six inches from the child's navel. A great effusion of blood followed, and the woman fainted. In this alarming situation Mr. B. determined on delivery by turning. On passing the hand up the vagina he found the orificium uteri in a lax and dilated state, and the shoulder presenting at the brim of the pelvis. By conveying the hand past the projecting part of the fœtus, he laid hold of its feet, and brought them down through the pelvis, whilst the shoulders receded backwards into the cavity of the uterus, thus accomplishing the delivery in a few minutes. The child appeared feeble, but soon recovered on being placed in a warm bath. A considerable hæmorrhage followed the birth, and therefore Mr. B. passed his hand into the uterus, and kept moving it therein, for a short time, till its contractions were renewed, when the hand was removed and the flooding abated. The woman soon recovered. We met with a case about seven years ago where the placenta presented, and was soon expelled, with very little hæmorrhage. The child followed in the course of one or two pains, and the woman lost very little more blood than in many cases of common labour. To a friend of ours, a similar case occurred; but the placenta and head were expelled by the same pain, and the body followed in two or three minutes without accident.

A few years ago our author met with a case of twins where several days intervened between the expulsion of the two placenta. The woman had been delivered during his absence, of two fine children. On his return next day, he found her restlesss, and labouring under much pain, with a slight hæmorrhage at intervals. An aperient draught, which operated thrice—an opiate at bed-time. Next day she was in the same state—he laid his hand on the abdomen, which appeared uniformly distended as high as the umbilicus, and rather tender on pressure. These symptoms led our author to examine, when, to his surprize, he found the *orificum uteri* wholly occupied with a placenta, which he readily extracted, a large gush of offensive coagula immediately following. The woman was much relieved by the change; but, for several succeeding days, there were occasional discharges of grumous blood from the uterus, accompanied with symptoms of puerperal fever, which disappeared in two or three weeks.

4. *Advantages and Disadvantages of inducing Premature Labour.* Cases but too often occur, where, owing to morbid structure of the female pelvis, parturition, at the full period of utero-gestation, cannot be accomplished without danger, either to the mother or child. If premature induction of labour can prove a substitute for embryulcia, humanity as well as policy would induce us to give it the preference.* The cruelty too often inflicted on the fœtus, by the crotchet, and the danger attendant on the mother, are sufficient reasons for preferring the process in question, with the view of preserving the life of the former, and lessening the risk of the latter.

"It is manifest that premature labour should never be attempted before it has been proved by the event of one or more destructive fœtal births, that the pelvis was so much distorted, that life must have been unavoidably sacrificed before delivery could be accomplished, because a single fatal instance is not always a sufficient warrant for the operation." 281.

---

* "Premature labour was first induced in this country, with success, by Dr. Macauly of London, about the year 1756."

It should be remembered that, in all cases where the head presents, (except in extreme degrees of distortion,) it is advisable to try what the powers of Nature will do, and never to have recourse to instruments till we are certain that she is inadequate to perform her office—there being few cases, (if the head be not absolutely too large and ossified, or the pelvis greatly distorted,) where the child may not be forced along and delivered with safety by the pains alone. By waiting in this manner, we gain a double advantage—first from the lengthening of the fœtal head; and next from its being forced low down into the pelvis, so as to render its extraction by instruments more safe and easy. It is evident that an accurate knowledge of the dimensions of a pelvis should be gained, before taking any decisive step on such occasions. The hand or fingers, Mr. B. justly observes, are preferable gaugers to all the *pelvimeters* that ever have been invented. When making this indispensable enquiry respecting the pelvis, and on passing the fore and middle fingers up the vagina, if the os coccyx, or any part of the sacrum or lumbar vertebræ, be perceived to project unusually into its cavity, or if the rami of the ischia approach so near each other as to admit no more space than two fingers to be placed edgewise betwixt them, we may conclude that, primary malformation exists in the bones which compose one or both of these apertures.

"Yet it not unfrequently happens that the brim of the pelvis is inconsiderably abridged, and the inferior aperture or outlet, is even wider than natural, and to obtain an accurate estimation of the contour of the two straits of the pelvis in every direction, when one or two fingers are inadequate for this purpose, it is necessary when admissible, to have recourse to the passing of the whole hand by the vagina into its concavity, the first three fingers of which are to be conducted to the brim, where the admeasurement may be generally ascertained, to the space of a few lines, by alternately placing them diagonally in the superior strait in the following manner:

"I.—If when the hand* is conveyed through the vagina to the brim of the pelvis extended, and the fingers kept close together, the side of the fore-finger touch the os pubis, and that of the little one, the projecting angle of the sacrum; the distance will be about three inches.

"II.—If on moving the little finger out of the way, and placing the other three conjunctively, in the same diagonal direction as before directed, and their sides come in contact with the pubis and promon-

---

"* A tolerable sized hand methodically directed up the vagina, will readily pass through the apertures of a pelvis, the small diameter of which does not exceed 2¾ inches from the pubis to the sacrum, even if the lateral dimensions are contracted."

tory of the sacrum; we may conclude that the space betwixt these two opposing points, is little more than 2 inches, an opening through which no mature fœtus can possibly be extracted alive, nor even if we suppose the space to be $2\frac{1}{2}$ inches.

"III.—When only two fingers can be placed edgewise in the fore-mentioned manner, and betwixt the two angles of the short aperture of the pelvis, the extent will not exceed $1\frac{1}{2}$ inches." 292.

From this last limited extent, the accoucheur may be assured that, when the period of gestation is completed, unless a greater distance can be obtained in either of the lateral diameters of the superior aperture, no other mode of delivery will be so safe for the mother, as the crotchet, or Cæsarian section. If the superior strait of the pelvis, from the symphisis pubis to the sacrum, or in any part from the anterior to the posterior point, exhibit a space for the reception of the head equal to three inches in diameter, we may not altogether abandon the hope of extracting a living mature child through such contracted limits, with either the forceps or lever—especially if the child's head be small, and the bones not too firmly ossified or connected by syneurosis, to move a little one on the other. On the contrary, when the pelvis is found not to exceed $2\frac{1}{2}$ inches from pubis to sacrum, or in any other direction, our author is persuaded, from long experience, that no mature fœtus can be extracted without inevitable destruction to the child, and great risk to the mother.

"It will be evident from these facts, that it is in this intermediate degree of distortion (comprising not more than a quarter of an inch) betwixt the possibility of effecting delivery without injury to the mother or fœtus with the *forceps* or *lever*, and that extent of deformity which requires the application of the *crotchet*, that premature delivery seems most likely to be advantageously produced. In such cases this method is preferable to the sacrifice of a mature fœtus by Embryulcia, whilst unavoidably endangering the life of the parent." 303.

When this operation (induction of premature labour) is determined on, we ought to select such time for its completion as may secure to the child every possible advantage for acquiring an uninterrupted birth—for the nearer the period of operation to that decreed by Nature, the greater the prospect of a successful issue. It has been proposed to excite premature delivery before the completion of the seventh month.

"But if we consider the puny condition of such ephemeral beings, and the misery to which their premature birth subjects them, we shall have little reason to prefer such a state to a mere nonentity, while the community derives no benefit from such precipitate proceedings." 311.*

It is well known that so long ago as the days of Hippocrates, it was supposed that a seventh month birth was more likely to do well than a fœtus born in the eighth month—and the truth of the observation is allowed by our author and by the generality of accoucheurs. But the cause of this curious phenomenon is not so easily ascertained. We shall indulge Mr. Barlow in his explanation, and in his own words.

"It is granted that there are more seven months children reared than those of eight, and daily observations sufficiently prove that abortions take place more frequently about the seventh month than at any other period of gestation. To account for this fact, it may be remarked, that there is comparatively a greater yielding of the cervix uteri at this time, than at the eighth month. The functions of the uterus manifest less resistance to the causes which oppose parturition, and its neck unfolds its contractions with greater facility. The fœtal head is also somewhat less, and parturition is rendered easier. During no stage of gestation does the uterus perform its parturient office with more irregularity than at this latter period: consequently the bulk and firmness of the fœtal cranium opposed to uterine resistance, and connected with a variety of casual and operative causes in severe and protracted labour, will unavoidably subject the fœtal head to a state of undue compression, and its neck sometimes to strangulations. These are accidents to which it would be less exposed at an earlier period of pregnancy under similar circumstances during its evolution from the womb. On these grounds we may account for the greater probability of rearing a premature fœtus of seven, than one at eight months. There is therefore no reason for believing any thing ominous in numbers; nor can I imagine any other method of elucidation, than what is thus supported by observation and experience." 319.

Here Mr. Barlow gives a short sketch of the history of premature birth, beginning, as usual, with the Bible, and quoting a passage from the second book of Esdras—"the women with child shall bring forth untimely children, of three or four months old, and they shall live and be raised up." Avicenna declares he has seen lively children born and reared in the sixth month. Brouzet, in his Essay on Medical Education, records an instance of a fœtus living at the early period of five months. The Mareschal Duc de

* The author has purposely avoided detailing the steps of inducing premature delivery, lest the knowledge of it should be applied to improper purposes. All professional men know that it merely consists in rupturing the membranes and discharging the liquor amnii.

Richelieu was born in the sixth month. Peu, Maubray, La Motte, and Van Swieten relate several examples of premature birth succeeding. The most modern and authentic instance is that recorded by Dr. Rodman in the Edinburgh Medical and Surgical Journal for 1815, where a child was born in the fifth month of utero-gestation, and successfully brought up. From our author's own observations he is convinced "that there are as many instances of successful births at the seventh as at the eighth month (we think more) owing probably to the causes before stated. Hence it is indispensably necessary for the accoucheur to bestow as much attention to procure living children in the former as in the latter period; and to adopt the mode of resuscitation in the early as in the more advanced births." Several cases have occurred in Mr. Barlow's practice, where re-animation has been effected after a lapse of half an hour from the time of birth. It may be remarked, en passant, that every induction of premature labour before the termination of the seventh month of pregnancy, can only have in view the safety of the mother. It can avail but little in preserving the child.

The *Sigultian* operation, or section of the symphysis pubis, is properly condemned by Mr. Barlow. It is of great consequence, he observes, in cases of distorted pelvis, to discriminate between exostosis and malacosteon, or mollities ossium. In the *latter* disease the bones will yield considerably, either by the introduction of the hand or the impulse of uterine action on the body of the child during labour acting like a wedge in forcing open the pelvic apertures. In Exostosis no such change is to be looked for.

"Eight cases of this species of progressive deformity have fallen under my notice; in one of which the projection of the last lumbar vertebra, at its union with the angle of the sacrum was so much bent forwards into the cavity of the pelvis, that, on the introduction of the fore-finger up the vagina, a protuberance was presented to the touch very much resembling the head of the fœtus, pretty far advanced into its cavity.* On carrying the finger a little higher anteriorly past the projection, I could with some difficulty ascertain the head of the child; but on moving it around, the distortion appeared so great, that the whole circumference did not exceed that of a half-crown piece.

"This occurrence was on the 29th of April, 1792, at which time I delivered the woman with the Crotchet, and the bones of the pelvis receded considerably to the impulsive efforts, during the extrac-

---

* "This protuberance had been mistaken for the presenting part of the child's head by the attending practitioner, who had, previously to my being called in, been in attendance two days and nights, and had determined on perforating the head with the scissars before my arrival at the patient's house."

tion of the head of the fœtus; yet, notwithstanding the flexibility of the bones of the pelvis, and the debilitated state of her constitution, she recovered speedily and without interruption." 329.

The distortion and structure of the bones of the pelvis, in the other women alluded to, bore such a similarity to those just described, that it is unnecessary to detail the circumstances respectively.

At page 335, our author queries how far it may be moral or just, to induce *early* premature labour, with the intent of saving the life of the mother and sacrificing that of the fœtus.

"The problem is not easily solved; the well known aptitude inherent in a woman to conceive, the consequent frequency of this occurrence in these situations, and the number of fœtuses eventually destroyed with the intent of sparing the life of the parent, are incidents of the utmost importance to the community, and claim a proportionate share of humanity and consideration from every accoucheur concerned on these unfortunate occasions." 335.

For our own parts, we profess that we should not be very squeamish upon these occasions. Where the life of the mother was in question, we should not hesitate, were we certain that there were twenty living fœtuses to be sacrificed, in order to save the parent. Till birth the fœtus is, in fact, but a branch or part of the mother, and that branch should instantly be sacrificed to save the trunk.

Since the year 1803, our author has been in the habit of frequently exciting premature labour, with success to the mother in every instance, and generally with safety to the child. As the cases bear a great affinity to one another, he has selected one case out of the number to serve as a specimen of the rest. Of this we shall present the particulars to our readers.

*Case.* Ellen Pickles, of Rishton, about three miles from Blackburn, had been afflicted with rickets in her youth, and was still of low stature. She had three children—the two first delivered by the crotchet, owing to a distortion of the pelvis—the life of the third being fortunately saved, and forming the subject of the following narrative. On the 15th January, 1803, Mr. B. ruptured the membranes, and desired her to dispatch a messenger to him as soon as she found labour pains coming on. In the evening of the following day, Mr. B. was summoned, and found the os uteri dilated to the size of a crown piece—the pains strong—the head of the fœtus moveable by pressure of the finger, consequently not fixed in the superior aperture of the pelvis. In two hours, the os uteri had completely dilated—the head of the child advanced some way in the superior strait, where it remained stationary for two hours longer, notwithstanding considerable uterine action. Thus situated, Mr. B was induced to apply the lever, lest the head of the child should suffer. In a few minutes, the birth was effected with tolerable ease, and perfect safety to mother and child. The period fixed upon in this case, was the latter end of the seventh or beginning of the eighth month, as nearly as could be ascertained. The child, when born, appeared lively but immature, and is now grown up. The mother died in a subsequent labour, after the use of the crotchet by another practitioner. 345.

Our author had taken accurate admeasurements repeatedly of this female's pelvis, by means of the hand, in the way recommended above, and uniformly found the superior aperture from pubis to sacrum, to measure rather more than $2\frac{1}{2}$ inches; and on both sides the space was also perceptibly diminished below this guage, the inferior aperture appearing somewhat less than natural.

The work before us closes with a synoptical table of the various degrees of distortion incident to the female pelvis, to which are annexed the different methods of delivery, as distinctive guides for the practitioner.

### THE SYNOPTICAL TABLE.

| | *The distance from the upper edge of the Symphysis Pubis, to the superior part of the Os Sacrum, or conjugate diameter of the Pelvis.* | |
|---|---|---|
| Well formed Pelvis. | From 5 to 4 inches. | Delivery by the efforts of nature alone. |
| I. First degree of deformed Pelvis. | From 4 to 3 or $2\frac{3}{4}$ inches. | Delivery by the efforts of nature, or assisted with the Forceps or Lever. |
| II. Second degree. | From $2\frac{3}{4}$ to $2\frac{1}{2}$ inches. | Premature Delivery. |
| III. Third degree. | From $2\frac{1}{2}$ to $1\frac{1}{2}$ inches. | Embryulcia, or Delivery with the Crotchet. |
| IV. Fourth degree. | From $1\frac{1}{2}$ inch to the lowest possible degree of distortion. | Cæsarean Operation. |

### *Successful Case of Cæsarian Operation.*

Formidable as is the operation of embryotomy, that of hysterotomy is far more so. Mr. Barlow has the honor of having performed this terrible operation with success—an honour which few, if any, of his countrymen can claim. This case has been published in the "*Medical Reports and Researches*" for 1798, and been alluded to by many obstetrical and other writers; but, of the present generation of surgeons, few are acquainted with the particulars. We shall, therefore, make them known here.

Jane Foster, of the village of Blackrod, was in her 40th year, of a robust constitution, and the mother of several living children, when she had the misfortune to fall from a loaded cart, the wheel of which passed over her pelvis as she lay on her back. By this accident, she was confined six weeks to her bed, attended by Mr. White, of Manchester, and others. It was supposed that much injury was done to the pelvis, besides fracture of one of the ilia. Soon after her recovery, she became pregnant, and on Friday, November, 22, 1793, she was seized with labour pains, being then at the full period of utero-gestation. She was attended on this occasion, by a midwife, who having waited some days, without prospect of delivery, called in professional assistance. On the 26th, our author saw the patient, in consultation with Mr. Hawarden, and on examining, per vaginam, was surprized to find, that he could barely pass his finger between the pubis and the last lumbar vertebra. Besides this, the outlet was so much contracted that he could hardly introduce three fingers at that part. With some difficulty, he carried up his finger sufficiently high to judge concerning the degree of dilatation of the os uteri, which appeared to be considerable, but no part of the child was within reach. Her pains had left her the night before—her anxiety was great—pulse full—respiration difficult, which symptom was moderated by the loss of ten ounces of blood. Delivery *per vias naturales* appearing impossible, the Cæsarian operation appeared the only alternative. On the following morning, the poor woman consented to the operation, which we shall give in the author's own words.

"The patient being taken out of bed, and placed upon a table, lying on her back, with her head raised by pillows, I began by making a longitudinal incision five inches and a half in length, as high as the navel parallel to the linea alba, and about two inches to the left of that line.

"The integuments and the left rectus muscle being cut through, a small opening was made through the peritoneum at the upper part; and by means of a probe-pointed bistoury, this membrane was dilated to the same extent as the external parts.* The uterus was now exposed to view and an incision of the same length was continued through it. The child presented with its breech, and was extracted through the artificial opening, but unfortunately was dead, yet did not shew any material signs of putrefaction. The placenta and membranes were then extracted with the greatest ease. The uterus was very thin, scarcely exceeding that of the peritoneum, and equally so through the whole extent of the incision. No attempt was made to examine the pelvis from the abdominal wound. The hands of a female assistant were applied on each side of the abdomen, to prevent the admission of external air, and to press out any blood that might be diffused among the intestines, after which the sides of the wound were brought together and secured by seven sutures, over which slips of adhesive plaster were applied, and the dressing completed by a few turns of a flannel bandage round the body.

"The peritoneum was not included in the sutures, and no part of the viscera protruded during the operation, neither were there any blood-vessels divided which required to be secured by ligature. It was a fortunate circumstance that no hæmorrhage followed the extraction of the placenta, as was to be apprehended from an atonic condition of the uterus, the effect of long distention. The womb contracted properly, the lochia were about the usual quantity, and continued as in other cases. The poor woman scarcely complained during the operation, so great was her fortitude. Soon after she was put into bed, she slept without taking any medicine for that purpose, and passed a good night. On the 29th she complained of a fulness about the region of the stomach, with an inclination to vomit, and on laying my hand on the abdomen, a degree of tension was distinguishable. Her tongue had a whitish appearance, and her pulse about 120. A laxative clyster was administered with the desired effect, and the painful tension of the abdomen yielded to the stimulating effects of a blistering plaster. In short, all the symptoms which had before indicated irritation, now suffered a very obvious remission. Four days having elapsed since the operation, it was thought eligible to remove every other suture; on the sixth the remaining ones were taken away, and the wound appeared perfectly healed.

"Though she had been a nurse to her other children, she experienced no uneasiness in her breasts on the present occasion. Her health continued in an improving condition until December 4th, when it received some interruption for a few days from a diarrhæa, but which was checked by an astringent mixture. On the 10th she ventured out of bed, on the 17th she began to attend to her domes-

---

* "It may be requisite to state, that at the commencement of the operation Mr. Hawarden was suddenly seized with a violent fit of syncope, which wholly incapacitated him from attending to the steps of the operation, and having no other professional person present, I was obliged to be assisted by a female attendant."

tic employment, from which time, to the present, (an interval of 28 years) she has enjoyed a good state of health, menstruated with regularity to the usual period of life, but never been pregnant." 361.

The other two cases having been unsuccessful, we shall not notice them here. The case quoted does infinite honour to the head, heart, and hand of the operator.

We have now extended our analysis rather farther than we originally intended. Mr. Barlow being more of a practitioner than a writer, has occasioned us some trouble, on that account, in analysing his production. When our author reasons, his language is too often diffuse, and his style not very clear. When he relates facts or observations, it is very much otherwise. The *matter*, however, being valuable, we are very willing to overlook the *manner* in which it is conveyed: and, upon the whole, the perusal of this work has left a very favourable impression on our minds, respecting the talents, the philanthropy, and the zeal of this veteran and experienced surgeon.

